# GeoFarmer: A monitoring and feedback system for agricultural development projects

**DOI:** 10.1016/j.compag.2019.01.049

**Published:** 2019-03

**Authors:** Anton Eitzinger, James Cock, Karl Atzmanstorfer, Claudia R. Binder, Peter Läderach, Osana Bonilla-Findji, Mona Bartling, Caroline Mwongera, Leo Zurita, Andy Jarvis

**Affiliations:** a CIAT International Center for Tropical Agriculture, Colombia; b Human Environmental Relations, LMU, University of Munich (LMU), Germany; c Department of Geoinformatics – Z_GIS, Doctoral College GIScience, University Salzburg, Austria; d CGIAR Research Program on Climate Change, Agriculture and Food Security (CCAFS), Colombia; e UNIGIS América Latina, Universidad San Francisco de Quito, Ecuador; f Laboratory for Human-Environment Relations in Urban Systems, IIE, ENAC, Ecole polytechnique fédérale de Lausanne (EPFL), Switzerland

**Keywords:** Digital agriculture, ICT, Interactive feedback, Geolocation, Monitoring, Evaluation

## Abstract

Farmers can manage their crops and farms better if they can communicate their experiences, both positive and negative, with each other and with experts. Digital agriculture using internet communication technology (ICT) may facilitate the sharing of experiences between farmers themselves and with experts and others interested in agriculture. ICT approaches in agriculture are, however, still out of the reach of many farmers. The reasons are lack of connectivity, missing capacity building and poor usability of ICT applications. We decided to tackle this problem through cost-effective, easy to use ICT approaches, based on infrastructure and services currently available to small-scale producers in developing areas. Working through a participatory design approach, we developed and tested a novel technology. GeoFarmer provides near real-time, two-way data flows that support processes of co-innovation in agricultural development projects. It can be used as a cost-effective ICT-based platform to monitor agricultural production systems with interactive feedback between the users, within pre-defined geographical domains. We tested GeoFarmer in four geographic domains associated with ongoing agricultural development projects in East and West Africa and Latin America. We demonstrate that GeoFarmer is a cost-effective means of providing and sharing opportune indicators of on-farm performance. It is a potentially useful tool that farmers and agricultural practitioners can use to manage their crops and farms better, reduce risk, increase productivity and improve their livelihoods.

## Highlights

• A geospatial cloud-based system GeoFarmer was designed and developed.
• GeoFarmer can be used as smart-monitoring system for agricultural projects.
• It provides tools for interactive feedback loops between platform users.
• Results and lessons learned from five pilots illustrate the flexibility of GeoFarmer.

## 1 Introduction

The agriculture paradigm is changing, with the collection and use of data for decision making becoming increasingly important (Pham and Stack, 2018). The strategic application of information and communication technologies (ICT) in order to improve information sharing has been used as one means to achieve economic growth and increase welfare in developing countries (Yonazi and Kelly, 2012 quoted by Baumüller, 2017). Smart farming (Griffith et al., 2013) using ICT components has been promoted by many national and international initiatives for inclusion in development initiatives (ARD, 2011). For scientists and agricultural practitioners, digital skills, including data collection methods, analytical techniques, and communication technologies, offer opportunities to understand complex farming ecosystems and to tackle the challenges of agriculture (Kamilaris et al., 2017). ICTs can provide farmers with better access to information and improve their ability to share knowledge amongst themselves and with others.

However, the use of ICT in agriculture does not always lead automatically to higher yields and profits for farmers. Even though ICT access and use are emerging fast in developing countries, barriers to accessing mobile-phone based agricultural services still exist (Aker and Mbiti, 2010, ARD, 2011). Though progress has been made through digitalization initiatives that lead to improvements for smallholder agriculture (Baumüller, 2015, Courtois and Subervie, 2014, Tata and McNamara, 2018), they still do not reach many farmers in developing countries. Lack of connectivity, missing digital capability and poor usability of ICT applications are some of the impediments that slow implementation of digital agriculture in the rural context (Baumüller, 2017, Salemink et al., 2015). If new solutions for digital agriculture do not address these shortcomings, farmers may face new digital poverty (May, 2012). ICT initiatives should recognize the local context of connectivity, users capacities, and the cultural background to avoid a digital divide with marginal groups of smallholders driven into digital poverty (Aker et al., 2016, May and Diga, 2015).

Despite the many barriers that still exist for employing ICT for agriculture, especially with marginalized communities in rural areas, mobile phone-based technologies are becoming increasingly important to close the last mile of communication. ICTs can ameliorate the lack of technical assistance and extension staff, and provide information to marginalized areas (Babu et al., 2015, Kiptot and Franzel, 2015). In recent years, ICT extension services, based on mobile phone services referred to as m-services, with the private and public sector working together often with limited personnel, have gained much attention. However, they often struggle to reach a level of sustainability and often do not fulfill their promised potential (Hatt et al., 2013, Wyche and Steinfield, 2016). Most information services focus on delivering information on prices, farming practices and weather (Aker, 2011, Tadesse and Bahiigwa, 2015). Few m-services offer training and extension services to farmers (Baumüller, 2017) and even fewer opportunities for farmers to share their experiences amongst themselves and with others.

Sharing experiences and information is crucial as farmers prefer to make their decisions based on discussions and their own experiences, rather than accept top-down generalized recommendations (Ingram, 2008, Wellard et al., 2013). Farmers’ preference for participating in the decision-making process changes the role of the extension agents: the extension technicians become catalysts, facilitators, and promoters of knowledge generation and exchange. These pluralistic extension systems are a key element of the shift toward Farmer-to-Farmer Extension (FFE). Their relevance is increasing, and they now complement traditional extension services (Kiptot and Franzel, 2015, Rao, 2007). ICTs can enhance dialogue and knowledge-sharing by farmers. Furthermore, ICTs can bring to scale these extension approaches based on local expert facilitators (LEF) and volunteer farmer trainers (VFT). Within this framework, younger members of the community who are more familiar with ICTs can play a major role in helping farmers access information through ICT (Muktar et al., 2015).

ICTs are not only important to improve extension services, but also to scientists who can use ICTs that facilitate interactions between them, experts and farmers. Farmers have the potential to provide massive amounts of useful data on their activities and experiences. ICT-based approaches are more cost-effective for data collection, monitoring and evaluation of agriculture development projects than traditional methods (Hammond et al., 2016, Jarvis et al., 2015). Thus, ICT-based solutions can play a major role in efficient data collection which can, in turn, be the basis for better decisions by farmers and policymakers (Delerce et al., 2016).

The advantages of digital agriculture are clear. However, to implement digital agriculture in the context of small farmers, we cannot simply throw ICT solutions at farmers: we need to design the solutions and development in partnership with farmers and facilitators in participatory projects.

In this paper, we first describe the design and development process of a modular ICT application system called GeoFarmer. Geofarmer was designed to provide a means by which farmers can communicate their experiences, both positive and negative, with each other and with experts and consequently better manage their crops and farms. We designed GeoFarmer in a collaborative, incremental and iterative process in which user needs and preferences were paramount. The aim was to get a customizable system for near real-time data flows between system users, i.e., experts to farmers, which could support processes of co-innovation and usage of GeoFarmer for citizen (farmer) science projects. We describe the iterative development process based on our experiences with GeoFarmer in five projects within four geographical domains in Tanzania, Uganda, Colombia, and Ghana. We present and discuss the results of the lessons learned from the five projects and indicate how GeoFarmer can be further developed and used to facilitate information and knowledge sharing amongst farmers and between farmers and scientists. Increased knowledge sharing can reduce the risk of failure through informed decision-making and improve the livelihoods of the small farmers.

## 2 Methods

The rationale of the GeoFarmer design process followed the Principles for Digital Development (Waugaman, 2016). Following these principles, the specifications for the design of GeoFarmer were defined as follows: i) employ a systems approach to design GeoFarmer and make it replicable and customizable in other countries and contexts; ii) develop a modular design, with a system that is interoperable with a well-documented Application Programming Interfaces (API); iii) use/modify/extend existing tools and follow open standards; iv) design and develop GeoFarmer in a collaborative, incremental and iterative process with inputs from diverse disciplines and constant reference to user needs v) document the design process, results and lessons learned throughout the development of GeoFarmer.

### 2.1 GeoFarmer design as a geospatial cloud-based system

**Fig. 1:**
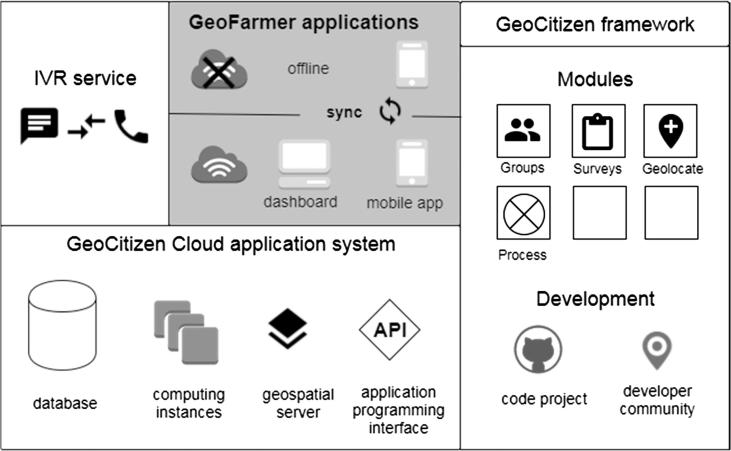
Overview of GeoFarmer application systems' architecture, developed as a subsystem of the GeoCitizen framework.

GeoFarmer uses a multilayer architecture with a system of modular components (functionalities and interfaces) that communicate with a central cloud application, which includes the central database where all information is compiled (see Fig. 1). The cloud applications’ backend also communicates with external components and services. The modular structure and multilayer architecture simplifies the development of single components for a specific usability context, like a simple user interface for standard users and a more complex interface for expert users.

We evaluated existing tools, platforms and frameworks to reuse existing approaches instead of developing new ones. These tools included several that have been developed and used for agricultural development projects. For example, the Open Data Kit (ODK) is widely used in development work in Africa, and others have integrated ODK into new applications. The Rural Household Multi-Indicator Survey (RHoMIS) uses ODK as survey module for a standardized and rapid characterization of households (Hammond et al., 2016). We considered incorporating ODK as a survey module in the early design process of GeoFarmer but found it challenging to integrate ODK in our system or interoperate between ODK and our database. Furthermore, it does not include two-way communication functionalities; hence we decided not to use ODK for the survey module.

From the evaluation process, we chose to develop GeoFarmer as a subsystem of GeoCitizen (Atzmanstorfer et al., 2014). The GeoCitizen framework provides several modules such as georeferenced surveys, geolocation of context-relevant information and structured and transparent discussion and feedback loops that fitted well with our aim of developing a system with near real-time, two-way data flows that support processes of co-innovation. Atzmanstorfer et al. (2014) developed the GeoCitizen framework to provide citizen participation in a structured manner with geospatial data collected from many sites, over time, by many participants, collated in a central database, and then interpreted by individuals and groups of citizens to meet their needs. The GeoCitizen platform has been applied for development projects in a long-term study in Ecuador, where it has been used in a participatory land-zoning process. Furthermore, GeoCitizen was subjected to a Human-Computer Interaction (HCI) evaluation study, carried out for the GeoCitizen-reporting application amongst members of marginalized communities in Cali, Colombia (Atzmanstorfer et al., 2016).

The backend of GeoCitizen provides application program interfaces (API) of functionalities that we used for user interfaces and applications. Features of GeoFarmer include data requests from the database and returning data for processing and storing. We used open-source component-based development frameworks for the cloud backend, web applications, and mobile application.

Existing modules and ICT components of GeoCitizen were adapted and modified for GeoFarmer to handle data and information in the context of agricultural development. We also added new complementary modules for GeoFarmer to the GeoCitizen application framework. We developed new user interfaces for GeoFarmer, which includes a smartphone application and a web-dashboard.

In recognition of low levels of ICT literacy frequently found in rural communities, where small farms are the norm, we emphasized simple, easy to learn functionalities. We developed a three-tier approach for farmers’ means of interaction with GeoFarmer to take into account the limited capacity for direct use by small farmers in some cases. First, user-direct second facilitated and third indirect.

### 2.2 Design as an iterative process to improve usability

The design and development team worked closely with scientists from various disciplines including computer science, geography, agriculture, and environmental change. The design and functionalities were improved in an iterative process from lessons learned in several pilot projects.

#### 2.2.1 GeoFarmer for evaluating agricultural best-practices in Tanzania

In a first pilot in 2014 and 2015, we examined the capacity of the GeoFarmer application system to support an ongoing citizen science project. Farmers in Lushoto, located in the Usambara Mountains in Northeastern Tanzania, co-managed demonstration plots with scientists and tested the effectiveness of climate-smart agricultural practices. GeoFamer was used to collect data and monitor the farmers’ uptake of and the effectiveness of management practices.

#### 2.2.2 Transect walks and repeating training with local youth facilitators

**Fig. 2:**
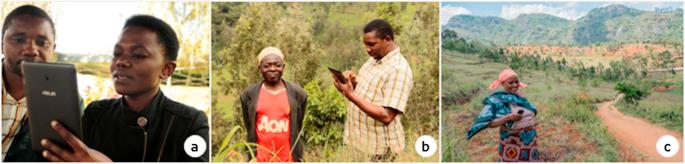
Youth facilitators from Lushoto during the training (a), a farmer responding to a survey carried out by a local facilitator (b) and (c), a farmer responding to a phone survey while being on the way to her field. Photo credit: Manon Koningstein (a,b) & Georgina Smith (c) / CIAT.

During the first pilot, future facilitators learned how to use the smartphone application of GeoFarmer. We trained three youth agricultural extension officers from Lushoto in two training sessions (Fig. 2). The objective of the first training session was to familiarise the facilitators with the basic functionalities of the system. The training focused on: i) registering farmers, ii) collecting face-to-face surveys with farmers and iii) collecting field points using the map functionalities.

We carried out transect walks with local experts, researchers and youth facilitators to gain experience on the use of GeoFarmer in the field. We collected observations on farming constraints, the crops farmers grow, topography, potential sites for demonstration sessions, and infrastructure such as schools for carrying out workshops with farmers. Observations from the training and transect walk on functionality and usability, i.e. youth facilitators requested translations of buttons and filters for registered farmers, were documented and used to improve the new versions of the application. At the end of the first training session, the youth facilitators used the application for several weeks, gaining experience that would provide feedback for the second training session.

In the second training, experiences with GeoFarmer were shared to provide insights on how it could be improved, i.e. participants mentioned the need for a offline functionality, and participants learned how to deal with more complex tasks, such as starting a discussion by publishing farmers’ observations and receiving comments from experts or other farmers on the map viewer.

### 2.3 The 5Q approach to monitoring progress through feedback

To set up an effective feedback mechanism between farmers and researchers related to project activities in the study area, we used the 5Q approach (Jarvis et al., 2015). The approach uses low-cost ICT tools to ask sets of five *smar*t questions to all stakeholders at regular intervals throughout the project cycle. “5Q approach moves from simply collecting data to using data from multiple sources to give a clearer idea of knowledge, attitudes, and skills” (Jarvis et al., 2015, p. 3) for a specific practice or technology to be evaluated for a specific geographical site. It uses *feedback rounds* as a new approach to monitoring the progress, and it uses different ICT components to collect information, i.e., it suggests using interactive voice response (IVR) surveys were possible and face-to-face surveys using ICT tools and the help of youth facilitators to complement the data gaps, and where the feasibility of phone surveys is restricted.

**Fig. 3:**
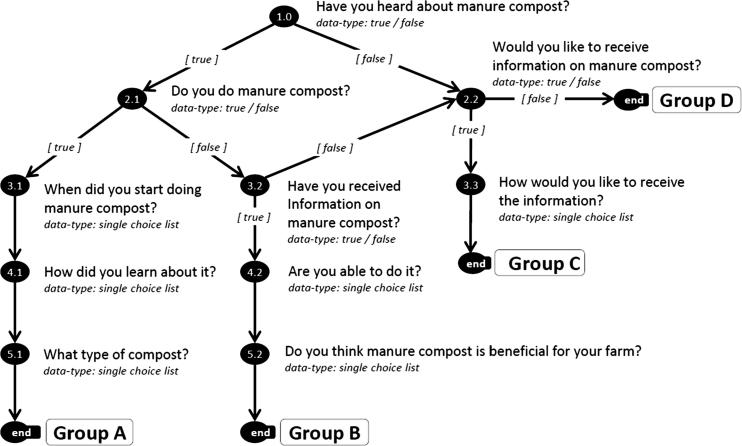
Question tree of the first round. In the end, farmers are grouped based on their responses.

In our first pilot in Tanzania, we experimented and compared the performance of 5Q IVR surveys and 5Q face-to-face surveys using the GeoFarmer smartphone-application by running them in parallel. We did this experiment after the second training session with youth facilitators. We ran feedback surveys with both, IVR calls and face-to-face surveys with registered farmers to monitor the uptake of climate-smart agriculture (Lipper et al., 2014) practices, i.e., farmers’ uptake of manure composting after demonstrations on farmer managed demonstration plots that have been operated throughout several months. We selected farmers for the IVR surveys based on the criteria of having own cell phones, which they do not share with others, and selected another group of farmers who did not have cell phones for the face-to-face surveys using the GeoFarmer smartphone-application. The question about having own cellphones was asked during the registration of farmers in GeoFarmer. We carried out two rounds of surveys with farmers in Lushoto with four months between the first and the second round of surveys. We designed surveys with questions trees (see an example for the first round in Fig. 3) using simple yes/no or single-choice questions.

In the first round, youth facilitators did face-to-face 5Q surveys with farmers using the smartphone application, and in parallel, we ran 5Q IVR call surveys on an external platform for mobile phone services (previously votomobile, now viamo). After finishing the first 5Q round, either on IVR or face-to-face surveys, farmers were grouped into typologies based on their answers. In the second round of surveys, we used distinct surveys for grouped farmers based on typologies from the first round.

### 2.4 Evolution of GeoFarmer through pilots in Uganda and Colombia

After experiences from the first pilot, we improved and tested GeoFarmer in pilot schemes in distinct geographic domains in Uganda, Colombia, and Ghana.

A pilot scheme was established in Nwoya, in the southern part of the Acholi sub-region in Northern Uganda in 2016 (Mwongera et al., 2016). Farmers participated in demonstration sessions on climate-smart agriculture practices similar to those in Lushoto. Our primary focus was to test the system in a different context, characterized by lower ICT literacy of the farming community and low availability of mobile data network coverage (internet access). A significant challenge to the functionality of GeoFarmer was the lack of mobile data network access. This difficulty was overcome by the development of an offline operating mode for GeoFarmer.

In 2016, we carried out a third pilot in Colombia. In this pilot, we focused on scaling the IVR calls and 5Q approach to 1240 farmers across the Province of Cauca, southwestern Colombia. We used IVR calls for collecting farmers’ perceptions of climate risks in the context of other risks that farmers face in agricultural activities. We used an existing database of farmers from Agronet (MinAgricultura, 2017) to carry out the 5Q IVR surveys.

### 2.5 Further development of GeoFarmer towards a SmartMonitoring system

In 2017, the CGIAR Research Program on Climate Change, Agriculture and Food Security (CCAFS) started using GeoFarmer to monitor and evaluate outcomes on its Climate-Smart Village (CSV) agricultural research for development (AR4D) approach, where CCAFS is testing since 2011 climate-smart agriculture practices with farmers, local experts from the national extension service and researchers alike. Together with the CCAFS team, we designed a set of indicators, related questions and survey blocks as modules that we tested in two additional pilots during 2017, in Colombia and Ghana.

We upgraded the development frameworks for the GeoFarmer mobile application and dashboard, using the latest backend for the GeoCitizen framework. Besides the latest technology, the main advantage of using the new framework was improved sync and on/offline functionality.

In Colombia, we examined the new GeoFarmer improvements in the CSV Cauca. We repeated training with facilitators before starting the survey data collection and carried out a small pilot of 60 farmers testing the new system. We used more extensive surveys with the focus on collecting data for tracking performance-based indicators on food security, climate services, practice adoption and among others. We faced the challenge of low mobile phone network coverage. The IVR calls did not work in this specific area of the Cauca department, and we had to do most surveys through facilitators.

Based on the experiences from Colombia, we decided to use the GeoFarmer smartphone application in offline mode and not the IVR calls for the next pilot in Ghana. Another reason for doing this, however, was the fact that our surveys covering indicators for CSVs were lengthy, complicated, and difficult to ask in IVR calls. For this reason, we combined registration, demographic baseline and several survey modules in one farm visit through local facilitators. The CSV in Northern Ghana, called Jirapa-Lawra, is situated in a landscape of Guinea Savannah woodland with low land productivity and distinct food security and agricultural adaptation strategies (Douxchamps et al., 2016). In this last pilot, we tested the system capacity of GeoFarmer and the new on/offline functionalities in a productive environment.

## 3 Results

We successfully designed and developed a prototype of an ICT applications system with near real-time, two-way data flows and the capacity to monitor processes of co-innovation in agricultural development projects. The design is a multilayers architecture with a system of modular components (functionalities and interfaces) that communicate with a central cloud application and can interoperate with external services, i.e., interactive voice response (IVR) services.

### 3.1 Specification of GeoFarmer

GeoFarmer was designed to house geospatial information and allows efficient feedback from and monitoring of farmers’ implementation of agricultural practices and technologies. Inputs to the system can be both directly online or via a specially developed smartphone application and alternatively through an interactive voice response (IVR) service.

For each geographical region or domain, GeoFarmer is modified to meet the specific requirements of that geographic domain, such as language, categories for data collection (crop species, production systems, used practices, and among others), predefined survey modules and map layers. Map layers are integrated as open standards such as Web-Map-Services (WMS). The geographical domains, wherever possible follow the idea of recommendation domains, which consist of farmers within an agroclimatic zone whose farms are similar and who use the same practices (Harrington and Tripp, 1984). Farmers and experts can add new content as georeferenced observation in the map viewer, including text descriptions, photos, and recordings. Moreover, they can add comments to existing observations of another user. In the following, we show a use case diagram and specify the functionality of different means of interaction.

#### 3.1.1 Use case diagram

**Fig. 4:**
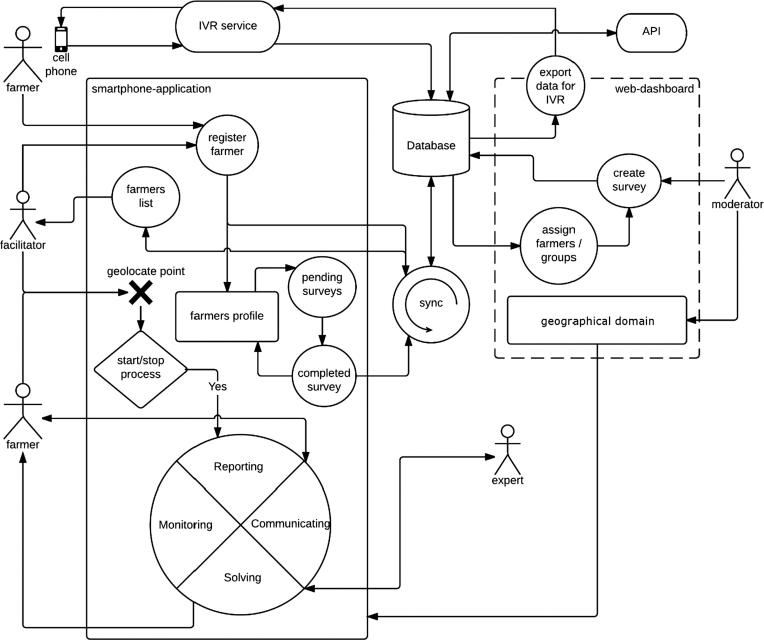
Use case diagram of GeoFarmer application systems, based on the GeoCitizen framework for citizen participation.

The use-case diagram in Fig. 4 provides an overview of the functionality of the GeoFarmer application system. The principal means of accessing the GeoFarmer application are (i) web dashboard, (ii) smartphone application, (iii) IVR calls and (iv) database. The GeoFarmer assigns distinct roles to four categories of users, (i) moderator, (ii) facilitator, (iii), expert and (iv) farmer. As many farmers either do not or cannot interact directly with modern ICT devices, facilitators act as catalysts either inputting data directly or helping farmers introduce data and information to GeoFarmer. The farmers, either on their own or with the facilitators, interact with the system through the smartphone application. The first step for farmers is to register. They can do this themselves or with the help of the facilitators. Once registered, the smartphone-application is used to collect point information and to participate in discussion processes with other farmers, facilitators, and experts. On the smartphone application, only the facilitators are authorized, through the system, to collect surveys with multiple farmers. Farmers and experts using the smartphone application can only view their information. All can participate in discussions. A moderator uses a web-dashboard to manage the system for a geographic domain and to organize surveys. The expert's role is to provide inputs to the discussion and answers to questions that have been uploaded by farmers or facilitators. Farmers can also interact and provide feedback through interactive voice response (IVR) services, which use automated phone calls to respond to surveys or to receive text and voice messages.

Following Atzmanstorfer et al. (2014), the system is based on processes. A single process consists of a discussion where the user can submit an observation or question. Other users (facilitators, experts, and farmers) can react to the observation and provide answers or vote for existing responses from other users. The system highlights best-voted responses as best practices and platform users can access the relevant information regarding this process. The conceptual idea comprises a social geoweb platform for sharing observations, discussing ideas, solving problems, and monitoring what farmers are doing. For the first version of GeoFarmer, we reduced the process functionalities to a simple *comment* function for users to make observations or comments. However, the GeoCitizen framework provides a more detailed process module, which includes discussion, voting, and rating mechanism to determine best practice solutions (see Fig. 4).

#### 3.1.2 Review of functionality

GeoFarmer systems functionalities allow users to interact with the system and carry out different tasks. Users with moderator role, i.e., local implementers of projects, use functionalities of overall project management mainly on the web dashboard. Users with facilitator role, i.e., project facilitators, support farmers in participating on two-way communication, and farmers as a user interacting by themselves with Geofarmer. Finally, users with expert role, i.e., agricultural scientists and extension technicians, use the smartphone application and web dashboard to contribute to knowledge sharing.

Table 1 summarises the systems functionalities, its objectives, user roles and means of interaction.

**Table 1:** The chart shows systems functionalities.

Systems functionality	Objective	Systems user and roles	Means of interaction
- User registration - Create geographical domain - Edit geographical domain	- Create a new user account - Create a new geographic domain, define the geographic extent, assign moderators - Edit domain parameters, define point categories, add map-layers, manage participants	Moderator (System Administrator)*	Web dashboard
- Approve user roles - New surveys - See/share survey results - Edit process parameters	- Approval of users as a facilitator/expert - Create surveys and assign surveys to farmer groups; create and add questions, edit survey parameters - Access and share survey results as a public link - Edit process parameters for the discussion process	Moderator	Web dashboard
- User registration - Register farmer	- Create a new user account - Register a farmer in the system	Farmer, Facilitator, Expert Facilitator	Smartphone-application
- Self-registration - List of farmers	- Self-registration of a farmer (profile) - Query/sort/filter list of registered farmers	Farmer Facilitator	Smartphone-application
- Edit Farmers (profile)	- Edit all farmers’ profile page - Edit own profile page	Facilitator Farmer	Smartphone-application
- Monitoring (surveys)	- Fill surveys assigned to multiple farmers - Fill surveys assigned to own profile	Facilitator Farmer	Smartphone-application
- Set a point-observation on the map - Communicating	- Geolocation of points on the map viewer - Start a participatory process on a point	Facilitator, Farmer	Smartphone-application
- Solving	- Comment, discuss, ask questions, provide answers - Users can vote (support) for answers	Experts, Farmer, Facilitators	Smartphone-application
- Monitoring (IVR)	- Run survey on IVR service portal - Respond to an IVR call of survey questions	*(System Administrator)* Farmer	IVR call
- Export data (for IVR calls) - Import data (from IVR calls)	- Export farmer lists, survey questions from the database - Import results from IVR service into the database	*(System Administrator)*	Database

* The system administrator is the platform operator.

In the following sections, we explain the four means of interaction in more detail.

#### 3.1.3 Web dashboard

The GeoFarmer dashboard is a management tool and integration platform for collecting data in the field. It is the central tool for managing GeoFarmer geographical domains and data. Only registered users with moderator role can log in to the dashboard and access their geographic domains (projects). The moderator creates new surveys and questions, and he approves facilitators that requested a facilitator role through the smartphone application. Collected survey data and results are accessible on the dashboard; the moderator can create public links of results and share them on the internet. The moderators manage the discussion process of smartphone-application users, i.e., set parameters or control user access to the discussion process thus ensuring a free exchange of information between users. Although the facilitators, experts, and farmers do not use the dashboard, their ability to communicate depends on it being well managed.

#### 3.1.4 Smartphone application

**Fig. 5:**
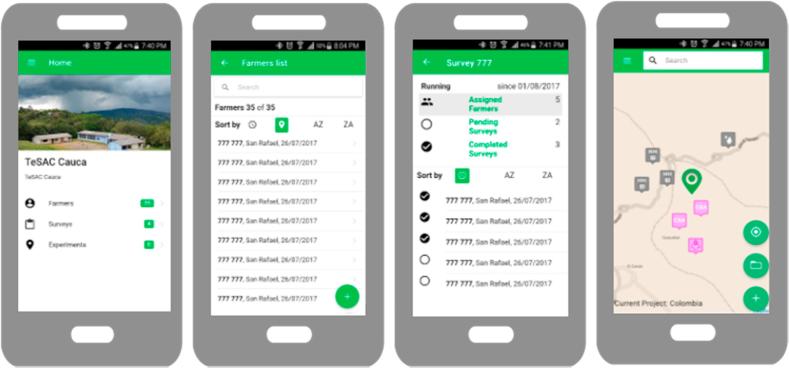
Selected screenshots of the smartphone application show the start page, the list of farmers’ page, a list of pending and completed surveys and the map viewer with observation points set by users.

Facilitators and experts use the smartphone application during fieldwork activities while interacting with farmers. Farmers can also use it as an individual user. It is the central data-collection tool (Fig. 5). The smartphone application is simple and optimized for fieldwork usage. After user registration and login, the user can send a request to be a facilitator in a specific geographic domain, which requires approval from the moderator in the web-dashboard, or he logs in as an individual user (farmer).

The facilitator can access the farmer's list and manage all registered farmers for the geographic domain. In an individual farmers’ profile, he can assign surveys that are available for the geographical domain to them. He registers new farmers in the menu *Farmers* by filling the farmers’ profile. The registration process includes a project-specific electronic consent statement in the farmers’ local language, which the facilitator must read to the farmer before finalizing the registration with the new farmer; the farmer must provide the electronic consent if he is to be part of the overall system. By clicking on the menu item *Surveys*, facilitators can access the list of available surveys for a geographic domain and search for farmers with pending surveys. Farmers logged in to the smartphone application can only access their surveys pending to be filled.

The user (facilitator, farmer, and expert) can collect spatial observations on the *Map viewer* page. The map viewer consists of simple GIS functionality for navigating (pan, zoom, GPS location) on a base map or geographical domain specific map layers, (which are added by the moderator in the web-dashboard). After setting a point on the map, the user can provide related information as text or media files and start a process assigned to the new location. When the smartphone is online, the server database and the local phone storage are synchronized. Once synchronized the smartphone application can be used offline. However, new data from other users up to the last time when smartphones were connected to the internet and synced with the central database, are not available while the application is in offline modus.

#### 3.1.5 IVR calls

Some of the farmers’ barriers to access to ICT technology in the developing world, like lack of smartphones, low ICT literacy and pluralism of local languages can be partly overcome by using voice-based channels of communication, such as call centers, voicemail or Interactive Voice Response (IVR) systems (Islam and Grönlund, 2010, Vashistha and Thies, 2012). IVR calls are a cost-effective alternative to collecting data compared to face-to-face surveys carried out by facilitators during fieldwork, and they are simpler to use by farmers than smartphone applications (Jarvis et al., 2015). We combined IVR calls with the existing survey modules adapted from the GeoCitizen framework to provide broader access to the GeoFarmer system. Currently, the IVR calls are not yet fully integrated into GeoFarmer. A third party platform (previously votomobile, now viamo) was used for IVR call surveys. The results of the IVR calls were imported to the GeoFarmer database.

#### 3.1.6 Database and backend functionalities

Database and backend functionalities are part of the GeoCitizen cloud-platform. Import/Export of data from the database requires a *systems administrator* role for the database and is carried out in this version of GeoFarmer through Standard Query Language (SQL) statements. In the next version, data import/export is planned to be integrated into the web-dashboard. The access to backend functionalities for the development of the GeoFarmer smartphone application is provided through an API and can be accessed by members of the GeoCitizen developer-community (see Fig. 1). Further developing the API of future versions of GeoFarmer could allow other applications to interoperate with the GeoFarmer database. Interoperability between ICT systems in agriculture is a crucial requirement for improving the sustainability of these systems.

### 3.2 Results and lessons learned from five pilots in four geographic domains

We tested GeoFarmer in four geographic domains associated with ongoing agricultural development projects in East and West Africa and Latin America. Our results demonstrate that GeoFarmer is a cost-effective means for data collection and potentially a useful tool that farmers and agricultural practitioners can use to manage their crops and farms better, reduce risk, increase productivity and improve their livelihoods.

#### 3.2.1 Experiences from testing the GeoFarmer and IVR surveys in Tanzania

Before we could start using the system in Lushoto, the moderator had to establish a geographical domain for Lushoto in the web dashboard. We also defined and configured categories for point-data collection. We used categories for crop cultivation, climate-smart agriculture practice, farm household, plant disease and point of interest among others. We added thematic map layers from existing map-services for the region to improve the cartography for the map-viewer. We included map layers of land-use classification, road network and main villages for the fieldwork. The moderator created the surveys on the web dashboard.

We used the smartphone application in transect walks with local experts, researchers and youth facilitators. The *map viewer* was opened in the menu option, and then the phones’ GPS signal provided the exact location on the map. Once the application received the position, the *collect* button was activated, and the location-specific information was entered. The entry consisted of a form to be filled in and the additions of photos taken by the smartphone camera and short descriptive text.

After the transect walks, the youth facilitators continued using the system between the first and second training. Over the six months from the first to the second training session, two local facilitators registered more than a thousand farmers from nearby villages and collected a baseline survey of demographic information with 91% of the farmers contacted. In total, facilitators registered 956 farmers with completed demographic surveys in GeoFarmer. Additionally, the two volunteers geo-referenced more than 670 field observations using the defined categories and provided data of cultivated crop species, farm locations and details of farmers’ field plots.

During the second training and transect walks we observed that the purpose of the more complex tasks of starting a process by commenting on others point-observations was difficult for local experts to practice. We noticed that it was necessary to repeat the basic tasks from the first training to improve local experts’ familiarity in using GeoFarmer. As a lesson learned from the second training, we concluded that more complex tasks need to be simplified and divided into simpler tasks and guided steps, with a focus on improved user experience and applications’ usability to carry them out.

**Fig. 6:**
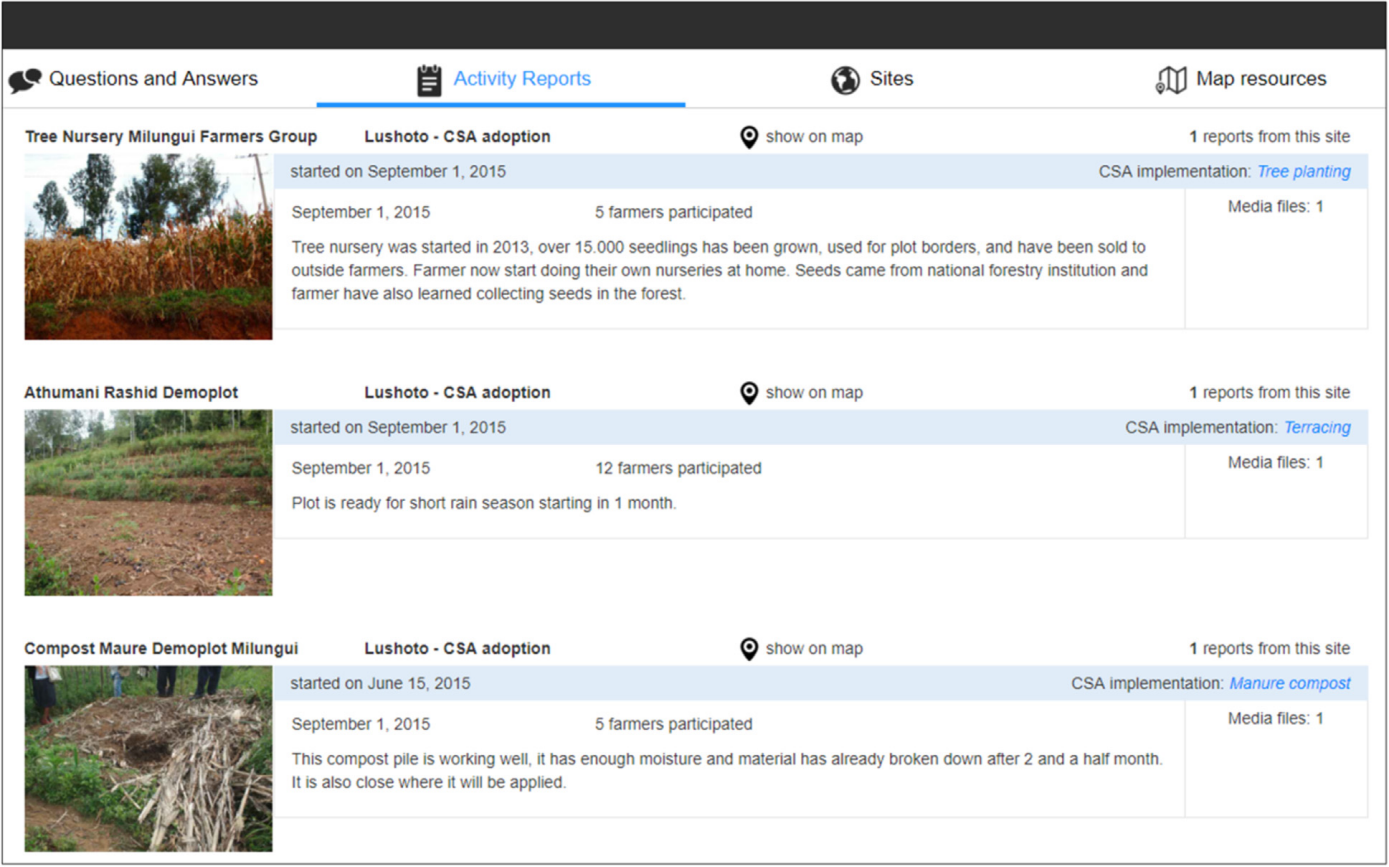
Screenshot showing uploaded information by facilitators to GeoFarmer web-dashboard.

After the second training with facilitators, however, we piloted Geofarmer testing the more complex tasks, such as publishing farmers’ observations, submit activity reports on demonstration plots and post questions of farmers. During the following weeks, facilitators published information from several demonstration plots by using the Geofarmer smartphone application (Fig. 6). The number of interactions with the system was still low in this first project phase, and it shows that facilitators had difficulties using GeoFarmer for information and knowledge sharing.

After the training, transect walks and registration of farmers in GeoFarmer, we started experimenting with using structured survey trees, following the 5Q approach, to obtain feedback from farmers on information provided to them on climate-smart agriculture practices. We compared the differences in cost-effectiveness, response rates and farmers preferences between face-to-face surveys through facilitators using the GeoFarmer smartphone application with that from the IVR calls.

**Fig. 7:**
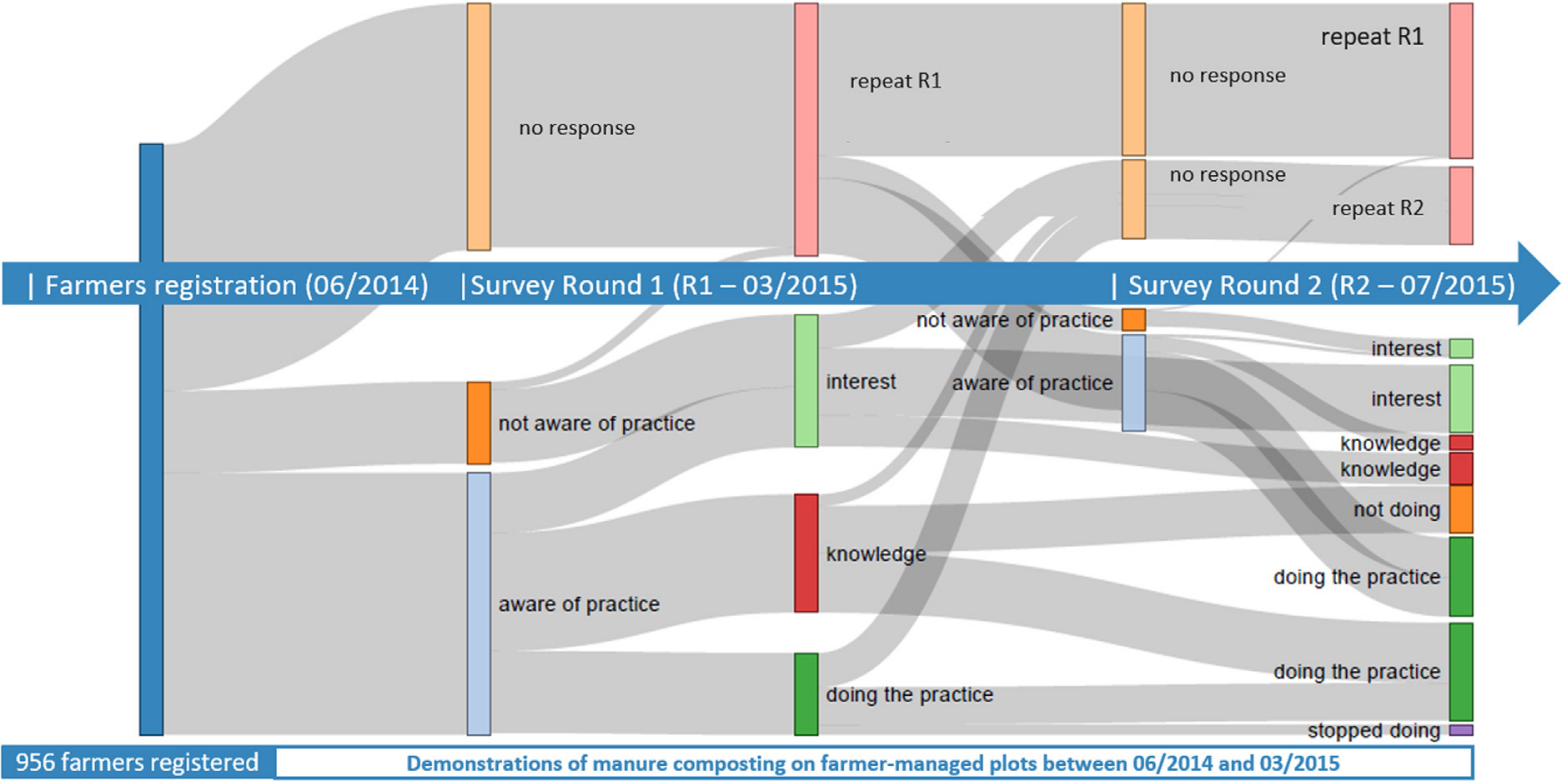
Farmers’ adoption of manure composting in Lushoto. The diagram shows the timeline of surveys from registered farmers in the first bar on the left (blue). Bars two and three show results from the first survey round, and bars four and five show results from the second survey round. At the end of each survey round (bars three and five), farmers are grouped based on their responses. The groups, in turn, determine the set of questions for the next survey round (see question tree in Fig. 3). Sankey diagram created with d3.js Sankey diagram http://bost.ocks.org/mike/sankey/. (For interpretation of the references to colour in this figure legend, the reader is referred to the web version of this article.)

Farmer’s adoption and awareness of manure composting were used to evaluate the effectiveness of the surveys. A series of surveys were initiated after the initial demonstration of manure composting to farmers in Lushoto. The surveys were carried out in six villages, surrounding the sites where the demonstration training was held, and where we registered farmers in GeoFarmer. The second round of the survey was carried out four months later, and it shows changes in farmers’ knowledge, attitudes, and skills about the climate-smart agriculture practice manure composting in Lushoto. We used a Sankey diagram to visualize the flow of information and awareness of the manure composting practices (Fig. 7). The chart shows registered farmers and timeline of surveys (blue bars) in the study area who were aware of the smart-practice manure composting (light blue bar), not aware (orange bar) and unsuccessful calls (light-orange bar). As a subset of aware farmers, it shows farmers practicing manure composting on their farm (dark green), farmers who know how to manure compost (red), and farmers who are interested in receiving more information on the practice (light green). The diagram shows the changes in farmers responses between the first (bar two and three) and second (bar four and five) survey. Between the two survey rounds, some farmers changed from doing manure composting to not doing it, some of them started manure composting and others maintained the same status as in the first survey.

**Fig. 8:**
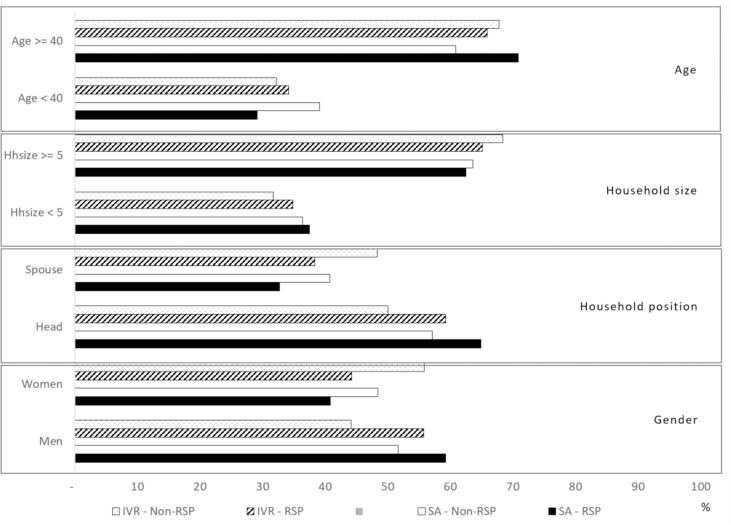
Demographic characteristics of Respondents (RSP) versus non-respondents (Non-RSP) for both, interactive voice response surveys (IVR) and smartphone application (SA).

We characterized respondents and non-respondents based on the demographic baseline that we collected when registering all farmers through the smartphone application. Fig. 8 shows the different demographic characteristics of age, household size, household position and gender of respondents (RSP) and non-respondents (Non-RSP) of both means of interaction. It shows that men are more likely to respond to both means of interaction than women are, and the heads of household have a higher share of respondents.

**Fig. 9:**
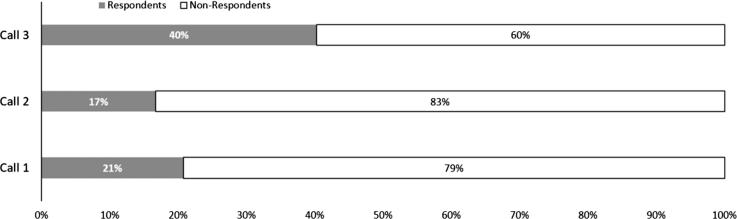
The response rate of farmers in Lushoto increased during three calls and applying several measures to improve the response rate.

We also found, from survey rounds where we tested different options, that the response rate of IVR calls in Lushoto depended on the way farmers were contacted. For example, both the time of the day when farmers were called and the prior announcement of the call, with information how the call was related to the project-specific participatory work and demonstrations, markedly influence the response rate (Fig. 9). The first two calls (Call 1, Call 2) were carried out between March and April 2015 with a response rate of 21% and 17% respectively. Social studies report response rates for IVR surveys of 20% to 30% (Dillman et al., 2009). For these calls, we did not inform farmers about the planned IVR calls, and we called them at any daytime (Call 1) and early in the morning as suggested by local experts (Call 2). Before Call 3, which was carried out in July 2015, we applied several measures to improve the response rate of farmers to the IVR surveys. The new approach consisted of three measures for improving the response rates on IVR calls. First, we announced the planned IVR surveys to farmers through our local project members. Second, we asked farmers about their preference of daytime for the call; they preferred in the late evening when they usually are back from their fields. Third, we sent a text message 30 min before the actual call. These three measures together increased the response rate to 40% in Call 3.

**Fig. 10:**
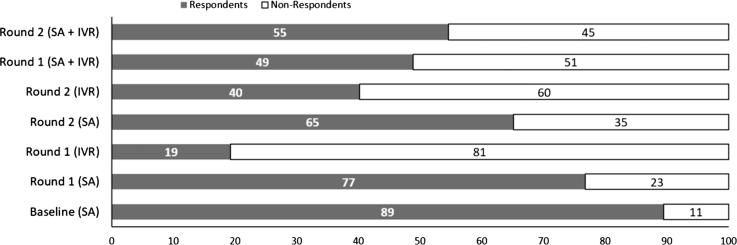
Comparison of response rate on smartphone application (SA) and interactive-voice-response (IVR) calls in Lushoto, showing two rounds of surveys (Round 1 and Round 2).

Fig. 10 compares the performance of face-to-face surveys using the GeoFarmer smartphone application (SA) carried out by the facilitators, and the IVR calls in Lushoto. It shows that the face-to-face response rate is better (between 65% and 89%) than the response rate from IVR calls (between 19% and 40%). The overall response rate of both methods face-to-face and IVR calls in 5Q round one and two in Lushoto were 49% and 55% respectively.

At the end of our pilot in Tanzania, we asked both facilitators and farmers if they found GeoFarmer to be a useful tool for carrying out surveys and collecting information. One of the facilitators in Lushoto said: *“Using the tablets, we can show pictures to farmers that we took on other farms, and the collection of surveys is more convenient using the tablets,”* Tanzania, June 2014. Farmer’s favored IVR call surveys, as one farmer in Lushoto during a field visit, said: *“It takes little of my time and I can attend the phone call anywhere, even when I am working on my field,”* (translated from Swahili, Tanzania, June 2015). The IVR surveys in Lushoto took approximately two to three minutes of their time for each survey round, and farmers could participate in them wherever they were and at almost any time. However, even with the improved measures doing the IVR surveys, the farmer response rate was lower as compared to face-to-face surveys that were carried out by the youth facilitators on the smartphone application.

#### 3.2.2 Experiences from testing IVR calls in Uganda and Colombia

Based on the Lushoto experience, we optimized the IVR surveys by first evaluating the local cultural context for operating phone calls with farmers, and we obtained response rates of 46% in Uganda and 43% in Colombia.

In Nwoya Uganda, despite the low mobile data coverage, with the offline capacity, we registered 355 farmers in GeoFarmer and carried out the IVR surveys. The questions in the surveys were in the Acholi language. The surveys were designed to obtain information on the adoption of smart agricultural practices. One hundred and sixty-four farmers answered the IVR surveys, and, 143 farmers listened to the complete introduction, 19 hung up before the introduction finished and 29 farmers did not pick up the phone. Farmers were asked questions about the *row-planting*, a practice which has been demonstrated previously in the region by the project. At the end of the IVR survey, we asked them to record their full name and confirm if they are male or female in a final question. We did this to verify the name with the name on the list of registered farmers in our database. Like in the first pilot, we organized the surveys as question trees of five questions following the 5Q approach, and we derived typologies of responses.

In the Cauca department in Colombia in 2016, we carried out one IVR survey including 1240 farmers across the Cauca department. At this time, using our experiences from previous IVR surveys, we achieved a 43% response rate on our first phone call to farmers. We did not use the GeoFarmer smartphone application in this pilot. We asked questions regarding farmers’ perception about climate risks in the context of other risks on agricultural production. Results show that 14% of farmers in Cauca are most worried about climate change and 28% perceive climate risks highest among other risks.

#### 3.2.3 Experiences from testing GeoFarmer in Ghana

During the year 2017, GeoFarmer was used to set up a comprehensive monitoring effort in the CCAFS CSVs. Together with the CCAFS team, we designed a set of indicators, related questions and survey blocks as modules that we tested in additional pilots during 2017, in Colombia and Ghana. Because of the length of survey modules, we decided to use the smartphone application for carrying out the surveys and did not use the IVR surveys. The offline mode was fundamental because of low internet connectivity in the study area. Ghana CSV was a first productive data collection where our youth facilitators collected more than 60.000 data records in offline mode from five survey modules during two weeks. They synchronized the data between server and smartphone application once a day when they had internet coverage through the mobile phone network, mostly when they finished their day and when they met at the main village.

## 4 Synthesis of lessons learned

In many developing countries, smartphone usage and internet coverage have increased significantly in recent years (Aker and Mbiti, 2010). Although the connectivity gap is expected to close shortly, last mile internet connectivity and lack of broadband access at village level is often a problem (Rao, 2007). We had functional internet connectivity in our first pilot in Tanzania, but experienced low connectivity in the other pilots. However, in all pilots, internet connection sometimes failed or was very unstable, and before we had implemented the synchronization mode, we were often not able to use the application. The offline and synchronization capability that was introduced primarily resolved this problem, as evidenced by the experience in Ghana with more than 60,000 records from 356 farmers collected in two weeks with poor internet access.

Another lesson learned is that digital illiteracy is a limitation for using ICT solutions in the context of small farmers in developing countries. Usability studies and the evaluation of Human-Computer Interaction (HCI) are an essential step in developing meaningful applications for users of marginalized communities with low ICT-skills. Within the GeoCitizen study, researchers carried out an in-depth HCI evaluation of its mobile app (Atzmanstorfer et al., 2016). We did not find it necessary to repeat this study for the GeoFarmer. However, observations of the facilitators during the training sessions, such as altering the visibility of buttons or reducing the number of steps to carry out a specific task, were incorporated into the application. Nevertheless, continuous improvement of the usability of the GeoFarmer application is necessary. Improvements can be mainly achieved by close interaction with the GeoFarmer developers and the facilitators.

In the pilots, there was a wide range of user types. Not all of the users were capable of using the GeoFarmer application. Facilitators were required to collect information. It is difficult to create a high-quality intuitive, easy to use app that can be used by the digitally semi-literate user but also has functionalities that have a high level of cognitive activity. This topic is still under-appreciated in the field of participatory tools, and further research in addressing user-friendliness and human-centered design approaches is needed (Çöltekin et al., 2010, Kramers, 2008). Developers of participatory tools mostly address the functionality of the system and the visualization of data and pay little attention to the user’s needs (Resch et al., 2014). Users’ needs are often left out the development process due to cost and time restrictions for analyzing the user’s needs (Watanabe et al., 2009). We suggest that future research and development of participatory ICT tools should take more into account user needs, preferences, skills, and capabilities, and focus on co-creation and co-development approaches for the design of ICT solutions. Applications should be improved so that more people can use them without the need for facilitators. Especially during our first pilot in Tanzania, we observed that the functionalities of more complex tasks like triggering a discussion-process by commenting on others point-observations, was difficult even for the facilitators. This aspect of usability needs further attention: too much attention is frequently given to providing information for researchers and not enough to how the farmers can perceive benefits from the sharing of knowledge.

Successful digital agriculture applications must take account of site-specific social and cultural differences. Furthermore, they are more likely to be adequately used if they form part of ongoing initiatives that have already gained farmers’ trust. For example, in the case of the first Colombia pilot, we worked through the partner Agronet, a Colombian agro advisory services initiative which farmers already knew and trusted. Agronet has been operational since 2005 to provide crop-related information to farmers. Most likely, because Agronet is well-known with farmers, the response rate on our first pilot with IVR surveys was high. Other studies in non-agriculture social science experiments reached 28% for IVR calls (Dillman et al., 2009), in our pilots, we first reached 17% and 21% in Tanzania and improved the response rate to 40% in the second round of IVR calls, 43% in the first Colombian pilot and 46% in Uganda. However, in the second pilot in Colombia, our experiences show that the farmers had little confidence in the phone-based surveys and response rates were low. Low cellphone connectivity in the area, with farmers lacking expertise with mobile telephones, appears to be the most likely cause of the flat response.

In agriculture research, the move towards two-way communication models between scientists and researchers and the lay population involves: (i) data on what is happening in the field (data capture); (ii) centralized databases and analysis of the data (data management and analysis) and (iii) interpretation of the information derived from the data analysis so that farmers can use it to make better-informed decisions (interpretation) (Cock et al., 2011). For the first pilot studies, we have used GeoFarmer mainly for data capture, and we have tested simple ways of two-way communication between farmers and agricultural practitioners. Another application of GeoFarmer could be in the field of citizen science.

Citizen science is based on establishing networks of non-scientists who participate and contribute to data collection and analysis of researcher-led projects. Citizen science makes science more inclusive, enabling scientists and citizens to co-create knowledge. The citizen science approach has been used by environmental researchers to allow the participation of large numbers of local stakeholders in initiatives addressing global change (Theobald et al., 2015, van Etten, 2011). Steinke et al. (2017) used a citizen science approach proposed by Van Etten (2011) for a farmer-managed variety selection trial in Honduras and showed that aggregated observations had sufficient validity. In such citizen science project, researchers are heavily involved in data capture and interpretation, with traditional researchers taking the leading role in data management and analysis, and the farmers and extension agents in charge of the interpretation and use of the information generated to make decisions.

Future research on ICT applications that enables two-way feedback and co-creation in citizen science projects should focus on improving usability and develop interfaces that are responsive to ICT literacy, like providing different user-experience and functionality for lay and expert users. A next version of GeoFarmer should integrate IVR functionalities in the systems’ API and use the different means of interaction context specific. For example, some farmers interacting themselves with the smartphone applications, others with the support of youth facilitators and in case of low internet connectivity or barriers of illiterately through IVR calls. Also, more research needs to be done to understand the barriers to and enablers for using such a system for information and knowledge sharing and in participatory citizen science projects.

## 5 Conclusion

Based on the premise that farmers can manage their crops and farms better if they can communicate their experiences, both positive and negative, with each other and with experts, we developed a tool GeoFarmer that expedites information sharing. We chose a digital system based on internet communication technology (ICT) as a cost-effective means for farmers to share experiences themselves and with experts and others interested in agriculture. During the development process we emphasized farmer participation in the design and testing of the system, GeoFarmer, so as to ensure both usability in areas with poor digital infrastructure and low levels of digital literacy and also that the overall system met farmers needs for information sharing, and the use of that information to make better decisions.

GeoFarmer is based on the GeoCitizen framework. The system comprises a multilayer architecture with modular components communicating with a central cloud application and database for safely storing and syncing data being sent from its components. It provides a sync-functionality for on/offline operation in rural areas with limited access to internet connectivity. The original GeoCitizen modules were adapted to characterize farming conditions and to collect and share experiences of small-scale farmers. GeoFarmer has to be tailored to each specific geographical domain and each of which requires a moderator. Trained facilitators ensure the participation of small-scale farmers with limited capacity to access or manage ICTs like smartphones. For data collections, IVR call functionalities complement the smartphone application. The design and development process of GeoFarmer was carried out in an iterative process from lessons learned in several pilot test sites, including scientists from different disciplines and feedback from users.

GeoFarmer was successfully used in five projects within four geographical domains in Tanzania, Uganda, Colombia, and Ghana. We used it to evaluate climate-smart agricultural practices on farmer managed demonstration plots in Tanzania and Uganda, designed as citizen science projects, and for monitoring and evaluation of indicators on outcomes of ongoing transdisciplinary research in CCAFS climate-smart villages. Results show the specifications of the developed system and experiences from testing GeoFarmer in the five projects. GeoFarmer was designed as a modular and customizable system for near real-term data flows between system users, i.e., experts to farmers and farmers to farmers, which support processes of co-innovation and can be used for Citizen Science projects in the agricultural sector. It allows efficient feedback from and monitoring of farmers’ implementation of agricultural practices and technologies.

Both facilitators and farmers found GeoFarmer to be a useful tool for carrying out surveys and collecting information. Farmers favored IVR call surveys as they took little of their time and were convenient when they were programmed in advance. However, the farmer response rate was weak when the mobile phone connectivity was poor, or, when we did not inform farmers and provide the context to a specific project activity before the IVR calls. There was a wide range of ICT capacity amongst the users. Facilitators widened the scope of users and enabled the inclusion of farmers with lower levels of digital literacy. However, future design and testing of Human-Computer Interfaces, like GeoFarmer, should include the participation of users with limited ICT skills, to prevent the need for facilitators. Also, currently IVR information from the IVR service is not readily transferred to the GeoFarmer API: data transfer between the two components needs to be improved.

This initial use of GeoFarmer indicates that it provides a means for farmers to communicate and share experiences interactively between themselves and with experts as they continually try new agricultural practices. We suggest that after this first step it can now be adapted and used for more comprehensive monitoring and evaluation of farmers’ attitudes and practices, and also to provide for farmers to share information and interchange ideas on how to better manage their crops and farms. However, the initial tests indicate that, even with facilitators, the feedback loops that form part of the discussion process with questions and answers shared between users’ needs to be further developed.
